# Supplementary material for: The interactome of intact mitochondria by cross-linking mass spectrometry provides evidence for coexisting respiratory supercomplexes
Source: Mol Cell Proteomics. 2017 Dec 8;17(2):216–32. doi: 10.1074/mcp.RA117.000470 (PMC5795388; doi:10.1074/mcp.RA117.000470)
Supplement: Supplemental Data [file supp_RA117.000470_133922_0_supp_23978_fzffwf.zip › spectra_annotation/mito_spectra_annotation/122-1-6-1-5-1.pdf]

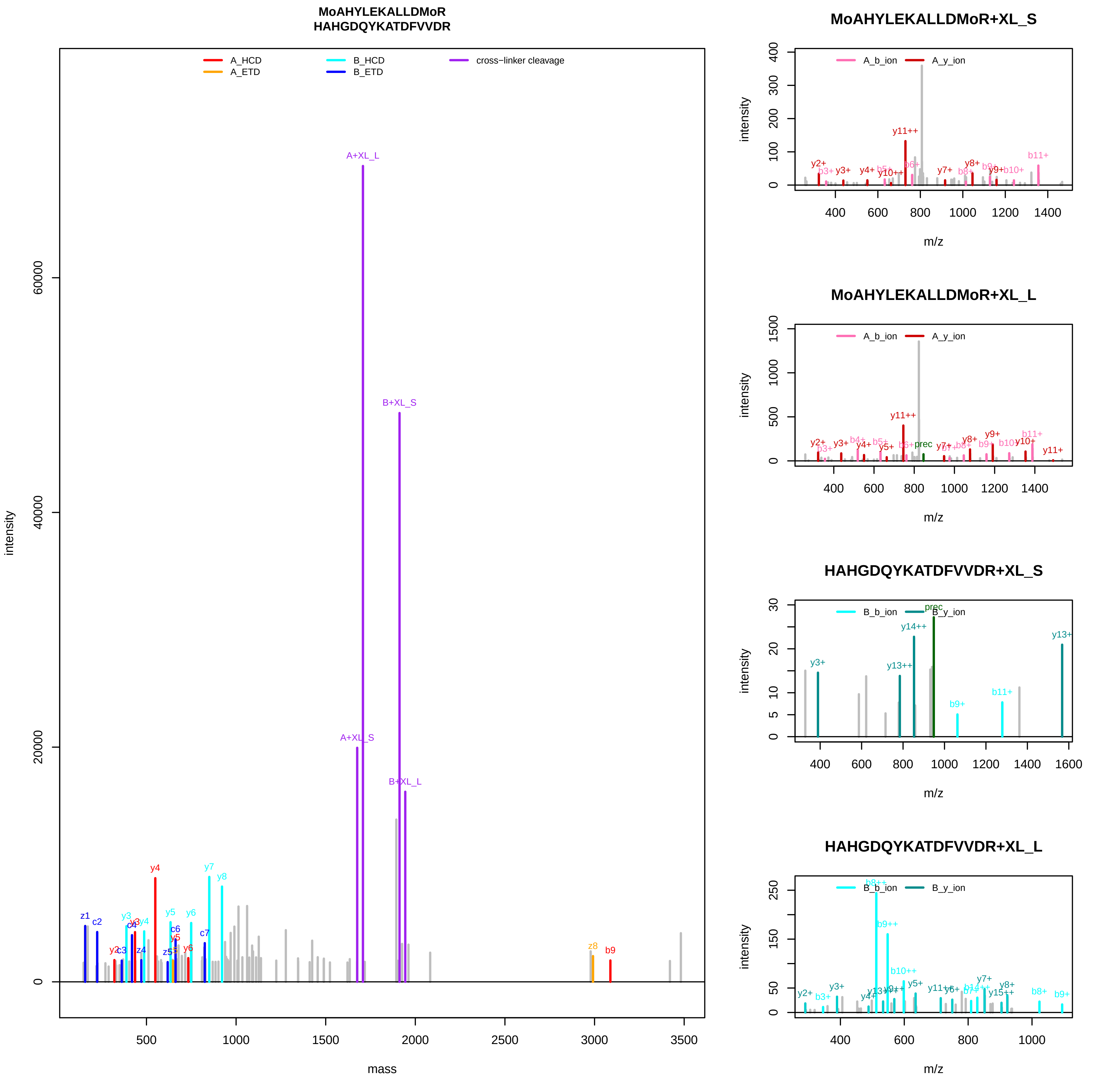
MoAHYLEKALLDMoR
HAHGDQYKATDFVVDR
A_HCD
A_ETD
B_HCD
B_ETD
cross−linker cleavage
A+XL_L
B+XL_S
A+XL_S
B+XL_L
z1
c2
y3
c4
y4
c3
y2
z4
y5
c6
z5
y6
c7
y7
y8
z8
b9
intensity
mass
MoAHYLEKALLDMoR+XL_S
A_b_ion
A_y_ion
y11++
y2+
b3+
y3+
y4+
y10++
b6+
y7+
y8+
b8+
y9+
b10+
b11+
m/z
MoAHYLEKALLDMoR+XL_L
A_b_ion
A_y_ion
y11++
b4+
b5+
y5+
prec
y9+
b10+
y10+
b11+
y11+
m/z
HAHGDQYKATDFVVDR+XL_S
B_b_ion
B_y_ion
prec
y14++
y3+
y13++
y13+
b9+
b11+
m/z
HAHGDQYKATDFVVDR+XL_L
B_b_ion
B_y_ion
b8++
b9++
b10++
y2+
b3+
y3+
y5+
y7+
y8+
y15++
b8+
b9+
m/z
